# A Quantitative Study of Early Childhood Care and Education Services Under the Age of Three: Evidence From Sichuan Province, China

**DOI:** 10.3389/fpubh.2022.794967

**Published:** 2022-02-11

**Authors:** Feng Peng, Xiaoyi Zhan, Bin Yang, Yihao Tian

**Affiliations:** ^1^Department of Public Service Management and Public Policy, School of Public Administration, Sichuan University, Chengdu, China; ^2^Social Development and Social Risk Control Research Center of Sichuan Philosophy and Social Sciences Key Research Base, Chengdu, China

**Keywords:** early childhood care and education services, qualitative method, demand, supply, Sichuan China

## Abstract

In China, early childhood care and education services for children under the age of three are neglected to some extent. Based on survey data from the Health Commission in Sichuan Province of China, this study analyzes the situation of early childhood care and education services in Sichuan through a qualitative method, using an analytical framework of matching the demand and supply. The findings reveal a mismatch between demand and supply of early childhood care and education services. This gap is likely to have a negative impact on children's self-development, family stability, and even the construction of national early childhood care and education service system. Drawing on the findings, suggestions for improving the mismatch between demand and supply are provided at the state, community and institution levels.

## Introduction

It has been widely accepted that early childhood care and education (ECCE) is significant for children's self-development, family stability, and even national construction. Almost all Organization for Economic Co-operation and Development (OECD) countries have explored their own models, forming the government-dominant models represented by Denmark and Sweden, the market-oriented models represented by the United States and Britain, and the multi-governance models represented by Japan and New Zealand (1–4). However, ECCE in China has been neglected to some extent; there is no mature model for ECCE. Traditional family rearing is still the principal approach China, especially by women, which has had a significant negative impact on the development of women. According to the survey report on the living conditions of working mothers in 2017, conducted by the Zhi Lian Zhao Pin, one of the largest employment platforms in China, 60% of women in the workplace believe that childbirth has an impact on their career development, 40% of women do not want to have children, and 36% of women have lost their jobs after giving birth, while this proportion was only 26% in 2016. With the development of China's economy and the implementation of the universal three-child policy, as well as the change in women's social status, the traditional model in China is no longer appropriate, and a more comprehensive method is needed to promote the improved growth of children, achieve improved parental development, and drive the improved construction of the country.

Previous studies have reached a consensus that ECCE plays an important role in child and mother development, family stability, and even national development. Effective ECCE services can ensure that young children receive good care, improve their intellectual development (5) and social cognition (6), and promote the overall development of children in the early stages of their lives so as to prepare them for school and lay an important foundation for their lives and learning (7). The research shows that the quality and enrollment rate of childcare are in direct proportion to the employment rate of women (8, 9); Thus, the optimization of ECCE can achieve improved work and family balance for women, especially working mothers (10–12). The emphasis on ECCE has the dual function of sustaining parental employment, especially for women, and fostering child development (5, 13). As for the country, the development of ECCE is an important means or tool to promote a country's human resources development (14), gender equality in the family and workplace (15–17), women's employment (18, 19), increase the fertility rate (20, 21), and reduce the intergenerational cycle of poverty (22). It has become one of the best investments a country can make in itself. Many countries and regions are increasingly strengthening the top-level design of a unified and perfected ECCE system so as to promote the co-development of children, family, and society (23).

The current research on Chinese ECCE can be divided into three aspects. The first is to emphasize the important responsibility of the government in the upbringing of children (24) and role orientation in promoting the construction and sustainable development of the infant care service system before the age of three (25). On this basis, some scholars have studied the development course of China's ECCE policy since the Reform and opening up of China (26), while others have provided policy suggestions for China through an international comparison of ECCE policies (27), particularly with welfare countries (28). The second aspect is to analyze the supply situation of ECCE in some developed areas, such as Nanjing (29) and Shanghai (30), and to identify, classify, and evaluate the current supply mode of ECCE for children aged three and younger in China (31). The third aspect researched the needs of parents (32), especially urban parents in developed areas (33). In summary, previous literature have focused on the policy, supply, and demand of ECCE, but there is a lack of systematic analysis of China's current ECCE situation from the perspective of matching supply and demand. Moreover, the research stays at the level of what the ECCE should be like and lacks effective data support.

In this context based on the survey data from the Health Commission of Sichuan Province, and by clarifying China's current ECCE policy background, we comprehensively evaluate the situation of ECCE in Sichuan province of China from the perspective of matching supply and demand, analyze the existing problems, explore the reasons for the current situation, and put forward feasible policy suggestions.

## Background

Due to economic development and social progress, more women have joined the labor market, which means that in China's dual income families, it is common for grandparents to be the main caregivers of children. Concurrent with family miniaturization and population aging, the change in the concept of childcare further refines and diversifies the requirements of childcare. The current method of family care cannot meet such requirements. Women's participation in the labor market also intensifies the problem of family care manpower. ECCE has become an important factor affecting children's and women's self-development, family stability, and even national construction (34). The construction of ECCE is helpful to solve the growing intergenerational gap caused by the unfair starting point of children, so as to better realize their own development. At the same time, it is helpful to ease the family care crisis to provide human capital support to help China escape the middle-income trap. In the context of China's universal two-child policy, it is necessary to establish an analytical framework for ECCE services with a widely accepted value criterion to understand the current domestic situation. According to The Convention on the Right of the Child and the literature review (35), the following will analyze the present situation of ECCE from the perspective of demand and supply. In order to do this, we must first understand China's current policy background.

### ECCE Policy in China

In China, the relevant policies on ECCE at the national level are constantly improving, especially after the implementation of the universal two-child policy; the pace of construction has accelerated, and policy attitudes have been clarified. The relevant policies at the national level are summarized in Table 1.

**Table 1:** Relevant ECCE policies at the national level in China.

**Years**	**Policies and regulations**	**Issuing department**	**Important content**
2010	≪Outline of the National Medium-and Long-Term Program for Education Reform and Development(2010-2020)≫	The State Council	Emphasize the importance of 0–3 years old infant education.
2011	≪Outline of Children's Development in China(2011–2020)≫	The State Council	Emphasize the development of public welfare and inclusive guidance institutions for children's comprehensive development, and provide early care and education guidance for 0–3 years old children and their families based on kindergartens and communities. Speed up the training of 0–3-year-old children's early education professionals.
2012	≪The 12th Five Year Plan for the Development of National Education≫	The Education Department	Strengthen the supervision of preschool education institutions and early education guidance institutions. Strengthen the scientific research of preschool education and promote the combination of preschool education and family education.
2012	≪Notice on the Pilot Project of ECCE for 053 years old infants≫	The Education Department	The pilot project of ECCE for 0–3 years old infants was carried out in 14 areas such as Shanghai and Haidian District of Beijing.
2014	≪National Development Plan for Children in Poor Areas (2014–2020)≫	The State Council	Carry out ECCE services for infants and young children. Provide ECCE guidance services for children under 3 years old and their families by relying on kindergartens and supporting education centers.
2019.5	≪Guidance on promoting the Development of Care Services for Infants under 3 Years Old≫	The State Council	Clearly put forward the basic principles of “family first, child care supplement” and “policy guidance, inclusive priority”. The main task is to strengthen the support and guidance for family infant care, increase the support for community infant care service, and standardize the development of various forms of infant care service institutions.
2019.10	≪Implementation Plan of Special Action to Support Social Forces to Develop Inclusive Care Services (Trial)≫	The National Development and Reform Commission; The National Health Commission	Carry out urban enterprise cooperation around “government guidance, multi-party participation, social operation, inclusive access”. Focus on supporting the construction of two types of nursery service facilities. The first is the demonstrative nurseries service institutions that undertake certain guiding functions; The second is the community care service facilities, which support the construction of a number of embedded, distributed and professional community care service facilities through new construction, reconstruction and expansion, and provide diversified inclusive care services such as full day care, half day care, temporary care, etc.

Central and local policies are highly unified in China. In addition to the relevant policies and regulations issued by the state, Sichuan Province has also issued some local policies and regulations, such as “the standard for basic kindergarten operation in Sichuan Province,” “the opinions on accelerating the development of nursery services for infants under the age of 3 years old,” and “the administrative measures for supporting kindergarten construction in residential areas in Sichuan Province.” There are three main tasks: First, scientific planning and construction of infant care institutions; second, the standardized development of infant care services; third, strengthening guidance services for infant family rearing. The scale, class size, staffing, and nature of the institutions are clearly defined. The introduction of these policies generally provides important guidance to promote the normative development of nurseries in Sichuan Province.

### ECCE Framework

#### ECCE Demand

ECCE demand refers to parents' desire to acquire ECCE services and the possibility of accessing them, which is important for parents, especially mothers, so that they can have time to work and receive professional nursery services. Accurately identifying demand is the basic premise required to meet demand. If governors cannot accurately grasp the demand for ECCE, they will be unable to identify those who have a strong need for ECCE and provide them with accurate public services; thus, there would be a large mismatch between demand and supply (36). In this process, we also need to pay attention to the various factors that affect requirements such as parental employment, income, education, and family structure (37), as well as the type and content of requirements, and so forth. Existing research to understand the real demand situation can mainly be categorized into three aspects of demand. The first is the total amount of ECCE, which represents whether people's demand for childcare services is strong. In this dimension, we will be able to see the overall demand for ECCE services. The second is the structure, which means that people prefer family ECCE services (which provide training services for parents or help parents take care of their children directly) or institutional ECCE services (services that can be accepted by sending children to institutions), and prefer public or market-oriented institutions. The third is characteristics, which refer to types of service hours, personnel quality, environmental facilities, and so forth. It is necessary to understand these aspects of demand, which are the basis for analyzing the matching of supply and demand.

#### ECCE Supply

ECCE supply means to protect the rights of the child and to meet the needs of the child and their family to receive special help for care and education, as well as all services provided and measures taken by the department concerned (this mainly refers to administrative authorities, public or private institutions, social sectors, and so forth).The generally accepted norm is that governments should provide ECCE services as public goods to all children and their families (19), especially high quality and inclusive services, or to assist market institutions in providing ECCE services as quasi-public goods. Theoretical researchers or practitioners have stressed that the insufficiency of ECCE services has a direct impact on the realization of children's rights (38) and can lead to other negative consequences such as gender discrimination, weakened family functions, declining social fertility (20) and labor market shortages (39). In view of this, the international community attaches greater importance to ECCE services for children aged three and younger, and mainly assesses the status of supply from the following three dimensions. The first is whether there is an overall quantitative balance between the supply and demand of ECCE services (40, 41); to some extent, this can be measured by more accurate statistics such as the rate of non-family childcare or the number of institutions (42). The second is whether the structure of ECCE services is reasonable. This refers to the proportion of ECCE services provided by government (43) or market-oriented institutions on the one hand, and the content of ECCE services on the other, which refers to whether the early care and education of children are taken into account and considered to be equally important. The third dimension is the quality of ECCE services (44), which includes safety and security (45), personnel quality (46, 47), service standards (48), and so forth.

As the fourth most populated province in China, Sichuan has the characteristics of a large population in urban areas, the rapid growth in the number of newborns, small family sizes, and an aging population. With the increase of migrants in the process of urbanization, the possible burden of ECCE in this province is incisive and vivid. Thus, we use the ECCE framework mentioned above to analyze the current situation of demand and supply in Sichuan Province.

### Methodology

There are 31 provinces in China. According to the data of the Seventh National Census, Sichuan Province has a permanent resident population of 83.675 million, ranking fifth in China and Sichuan's GDP is 4,859.88 billion yuan, ranking sixth in China in 2020. Meanwhile, multi-ethnic gather, covering large, medium and small cities, which can represent China's development to a certain extent. Since 2010, there have been more than 700,000 newborns annually in Sichuan. The number of newborns in 2017 was the highest at 930,000. To understand the ECCE status of the early lives of children in the province, the Health Committee in 2020 conducted a large-scale social survey in all cities in the province, covering 90% of county-level administrative units. This survey is the first provincial survey conducted by the Sichuan Provincial Health Commission after the implementation of the ECCE policy, and it is a random sample of cities in Sichuan Province according to the proportion of population. It has the largest scale, widest scope, and largest number of samples nationwide; thus, it is typical and representative. The survey contents can be divided into two parts. The first part is a large-scale population sample survey for families with children of preschool age. A total of 49,838 questionnaires were distributed, and invalid questionnaires with blank and logical errors were excluded. As a result, the remaining 44,540 questionnaires were collected, and the recovery rate was 89.40%. The second part is large-scale census data for ECCE institutions, which surveyed thousands of ECCE institutions in 21 prefecture-level cities or autonomous prefectures. The contents of the survey involve basic information about family members, family needs for early childhood education and care, general information about ECCE institutions, and so forth. It has good representativeness and research value. Therefore, this study uses the survey database to analyze the demand and supply of early child education and care in Sichuan Province. Next, it will be divided into three dimensions, namely, Sichuan Province's city profile, basic household survey information, and basic institutional survey information, to describe the social survey database of the Health Committee.

There are 21 municipal administrative regions in Sichuan Province. Chengdu is the capital of Sichuan Province, and Aba, Ganzi, and Liangshan Autonomous Prefectures are ethnic minority communities. Detailed descriptions of cities are provided in Table 2.

**Table 2:** Basic situation of cities (in 2018).

**City**	**Resident population(10,000 persons)**	**GDP(100 million yuan)**	**Urbanization rate(%)**	**Number of kindergartens**	**Number of health institutions**
Chengdu	1633	15,343	73.12	2,528	10,755
Zigong	292	1,407	52.61	429	2,245
Panzhihua	124	1,174	66.59	194	1,056
Luzhou	432	1,695	50.46	723	4,616
Deyang	355	2,214	52.35	337	2,819
Mianyang	486	2,304	52.53	792	4,674
Guangyuan	267	802	45.63	308	3,540
Suining	320	1,221	50.02	512	3,779
Neijiang	370	1,412	49.10	673	3,297
Leshan	327	1,615	51.83	732	3,259
Nanchong	644	2,006	48.14	793	8,583
Meishan	298	2,026	46.32	928	5,260
Yibin	456	1,250	49.64	688	3,446
Guangan	324	1,690	41.86	741	4,293
Dazhou	572	1,067	45.52	748	3,438
Yaan	154	1,256	46.85	454	2,040
Bazhong	332	646	41.85	260	3,274
Ziyang	251	646	42.71	268	1,456
Aba	94	307	40.00	308	1,683
Ganzi	120	291	31.66	400	2,777
Liangshan	491	1,533	35.71	580	5,249

Before reporting the basic information of parents and their children in the database, this study performed some data cleaning work. Samples with children over the age of three were deleted to ensure the representativeness of the sample. Meanwhile, we excluded samples with many missing values. The final sample size is 40,087.

From the perspective of the gender of the children, the male to female ratio is roughly balanced. From the perspective of age, most of the parents (about 70%) are between the ages of 25–35. From the perspective of salary, the average monthly income of parents is about 5,600 RMB(¥), which is almost the same as the average monthly income of 5,400 RMB(¥) in Sichuan Province. Detailed descriptions of families are provided in Table 3.

**Table 3:** Basic information of families.

**Variable**	**Label**	**Frequency**	**Percent**
Age of children	0–1 years old	10,313	25.73
	1–2 years old	13,723	34.23
	2–3 years old	16,051	40.04
Gender of children	Male	19,994	49.88
	Female	20,093	50.12
Age of parents	Under 25 years old	6,614	8.25
	25–30 years old	27,800	34.68
	31–35 years old	29,122	36.32
	36–40 years old	11,304	14.10
	Over 40 years old	5,332	6.65
Income of parents	0–1,500	5,031	6.28
	1,500–3,000	10,072	12.56
	3,001–5000	23,991	29.92
	5,001–8,000	19,702	24.57
	Over 8,000	21,376	26.66

In order to understand the current situation of the provision of childcare services in the early lives of children in the province, the Health Committee organized this survey. The basic information about ECCE institutions mainly includes the total number of institutions, types of institutions, business locations, investment status, and so forth. In addition, this survey also collected supply-side information on the changing situation and the number of children that can be taken care of.

## Results

Based on the data from the Health Committee, this study analyzes the current situation in Sichuan from the perspective of ECCE supply and demand. Specifically, according to our analysis framework, we will first study ECCE demand from the perspectives of the total amount, structure, and characteristics.

### The Current Situation of ECCE Demand

According to our analysis framework, ECCE demand mainly includes three aspects, namely, total amount, structure, and characteristics.

First, the total demand for ECCE services in Sichuan Province is strong. According to the data, 57.13% of respondents expressed their willingness to allow their children to receive ECCE services. From the perspective of “Hukou” or household registration system which was established in the 1950s in China and was primarily intended to achieve social control of population mobility and resource redistribution. It classifies all citizens as rural and urban. The ratio of urban Hukou for children under the age of three who choose ECCE is 5.30% higher than that of rural Hukou. From the perspective of residence, the proportion of families that choose ECCE services has gradually increased from 49.16 to 63.75% from rural areas to suburbs and urban areas. From the perspective of minority areas, the proportion of families in non-minority areas who choose ECCE is 10.70% higher than that in minority areas. Detailed descriptions of willingness to demand ECCE services are provided in Table 4. YES means choosing or accepting ECCE services, NO means being unwilling to choose or rejecting ECCE services. YES% and NO% represent the percentage of the corresponding population (YES and NO in subsequent tables also have the same meaning).

**Table 4:** Total demand for ECEC.

**Variable**	**Label**	**Yes**	**No**	**Yes %**	**No %**
Hukou registration	Urban registration	8,772	5,806	58.83	38.94
	Rural registration	13,165	11,430	53.53	46.47
	Other	487	427	53.28	46.72
Residence	Urban	6,164	3,505	63.75	36.25
	Township	3,058	1,866	62.10	37.90
	Suburb	8,355	6,984	54.47	45.53
	Rural	4,708	4,869	49.16	50.84
	Other	336	242	58.13	41.87
Ethnic areas	Non-minority area	21,375	15,641	57.75	42.25
	Minority areas	1,445	1,626	47.05	52.95

*Other options include all options except those listed (the same as below)*.

It can be seen that people with urban Hukou who live in cities are more willing to choose ECCE services. On the one hand, this is because having urban Hukou means that you will receive more public services, so it is possible to bear the cost of receiving ECCE services. On the other hand, people who live in cities often have no time to take care of their children because they have to work. Respondents from non-ethnic areas are more willing to choose ECCE services than those from minority areas. This may be because the economy in minority areas is underdeveloped and there is no such need. However, it may be due to insufficient emphasis on children's education in the region.

Second, the demand for ECCE has an obvious structural division between service providers and service content. As shown in Table 5, from the perspective of the nature of service providers, people are more inclined to choose public institutions such as public kindergartens. The proportion of people who chose public kindergartens is 82.78%, and the proportion of people who chose private kindergartens and commercial early education institutions are 33.99 and 51.71%, respectively. From the perspective of service content, there is no significant difference in the proportion of Sichuan Province's demand for household ECCE services and institutional ECCE services. The proportion of the population who prefer institutional ECCE services is 52.30%, and the proportion of the population who prefer family ECCE services is 47.70%. Thus, 32.56% prefer education, 28.64% prefer nursing, and 65.73% prefer nursing and education.

**Table 5:** Structure of demand for ECEC.

**Variable**	**Label**	**Yes**	**No**	**Yes%**	**No%**
Service provider	Public kindergartens	33,184	6,903	82.78	17.22
	Private kindergarten	13,626	26,461	33.99	66.01
	Maternal and child health hospital	17,334	22,753	43.24	56.76
	Commercial early education institutions	20,729	19,358	51.71	48.29
	Community nursery	17,049	23,038	42.53	57.47
Content	Family ECEC	19,121	20,966	47.70	52.30
	Institution ECEC	20,966	19,121	52.30	47.70
Preference	Education	13,052	27,035	32.56	67.44
	Care	11,481	28,606	28.64	71.36
	Both	26,349	13,738	65.73	34.27

Third, the types of requirements are complex. The complexity of the types of needs is mainly reflected in the cost, care methods, and anxiety, as shown in Table 6. From the cost point of view, 55.10% of families can pay nursery fees of 1,000 yuan or less, 35.41% of families can pay nursery fees of 1,001–3,000 yuan, and 9.49% of families can pay nursery fees of 3,001–5,000 yuan. Thus, the current average acceptable ECCE cost is about 1,300 yuan. From the perspective of the method of care, 72.88% of families choose full-time care, which is the dominant position. However, 19.47% of families still choose part-time care. This shows that families' current care power is scarce. From the perspective of anxiety, there are three main points. The first is that parents are worried that it will be difficult for institutions to take care of young children. The second is that the cost is too high for the family to afford it. The third is that parents are too busy at work and there are no grandparents living in the home; thus, there is no one that can take care of the children at home. The proportion of people who chose the above three points accounted for 69.48%, 69.47% and 49.44%, respectively.

**Table 6:** Characteristics of demand for ECEC.

**Variable**	**Label**	**Yes**	**No**	**Yes%**	**No%**
Accept cost	0–10,00	22,088	17,999	55.10	44.90
	1,001–3,000	14,195	25,892	35.41	64.59
	3,001–5,000	3,804	36,283	9.49	90.51
Care type	Full time	29,215	10,872	72.88	27.12
	Part time	7,805	32,282	19.47	80.53
Anxiety	Low age	27,852	12,235	69.48	30.52
	Expensive	27,848	12,239	69.47	30.53
	No time	19,819	20,268	49.44	50.56

In summation, the lack of potential caregivers at home is the most important motivation for choosing ECCE services, followed by the hope that the child will receive a better education. The poor economic status of the family and the concern that institutions cannot take care of very young children are the main obstacles to reducing the willingness to make use of ECCE services.

### The Current Situation of ECCE Supply

ECCE supply refers to all services provided and all measures taken by the public sector, market institutions, or the third sector to protect children's rights and meet the special needs of children and their families in care and education. Based on the survey data of the Health Committee of Sichuan Province, this study performs a detailed analysis of the current situation of ECCE services in Sichuan Province. The findings are as follows.

First, from the number of childcare institutions, the number of service opportunities that they can provide for children, the number of ECCE opportunities that are already in use, and the number of ECCE employees in different cities of Sichuan Province, we can form a general picture of the total supply. In 2020, there were 6,883 ECCE institutions in Sichuan Province that provided childcare services for children under the age of three. Chengdu was the city with the largest number of ECCE institutions, which accounts for 22% of the total. The city with the lowest number of childcare institutions is Ganzi, which accounts for 0.20% of the total (other cities are shown in Table 7). Second, all institutions in the province can provide 271,420 ECCE service opportunities at present, and the number of children already enrolled is 148,739. From the perspective of specific cities, Chengdu provided 64,798 ECCE service opportunities, accounting for 24% of the total supply. The lowest number of ECCE service opportunities was 353, and they were provided by Ganzi and accounts for 0.13% of the total. The average ratio of the number of service opportunities that are being used to the number of service opportunities that are supplied is 54.80%; Leshan has the highest enrollment rate (89.65%), and Meishan (78.55%) and Suining (76.51%) follow closely (other cities are shown in Table 7). Third, the number of full-time ECCE employees in the province is 84,910. The city with the largest number of employees is Chengdu with a total of 22,403 full-time employees, and the city with the lowest number Ganzi with only 50 full-time employees (other cities are shown in Table 7).

**Table 7:** The number of childcare service institutions, the number of available ECCE opportunities, the number of enrolments, and the number of employees in each city.

**City**	**Number of institutions**	**Number of available ECCE opportunities**	**Number of enrollment**	**Proportion of enrolment number to supply number (%)**	**Number of full-time employees**
Chengdu	1,538	64,798	36,341	56.08	22,403
Dazhou	675	30,085	17,266	57.39	8,154
Mianyang	567	27,824	11,106	39.92	7,442
Neijiang	417	11,765	5,434	46.18	3,924
Guang'an	388	13,230	6,425	48.56	2,442
Suining	375	14,965	11,451	76.51	5,197
Meishan	372	14,740	11,578	78.55	5,043
Yibin	358	11,664	4,902	42.02	4,408
Nanchong	353	16,894	7,575	44.83	4,233
Guangyuan	298	11,050	7,097	64.22	3,278
Deyang	227	11,716	6,508	55.54	2,177
Ziyang	226	7,844	3,244	41.35	2,469
Ya'an	224	6,336	3,889	61.31	3,235
Zigong	214	5,659	2,864	50.60	2,686
Leshan	198	6,436	5,761	89.65	2,021
Luzhou	110	4,857	1,942	39.38	1,125
Liangshan	107	3,163	1,581	49.98	1,050
Panzhihua	94	3,659	1,613	45.19	1,340
Bazhong	79	3,319	1,379	41.54	1,551
Aba	52	1,063	607	57.10	682
Ganzi	11	353	176	49.85	50
Total	6,883	271,420	148,739	54.80	84,910

Regarding the structure of the supply, there is not only a shortage of public ECCE institutions, but also a lack of care institutions that provide simultaneous infant care and education services. Market-oriented institutions provide more ECCE products and services. Among the 6,883 institutions surveyed, only 17.86% are public nurseries. Regarding the remaining 82.14% of non-public nurseries, 3,370 are profitable nurseries, accounting for 48.44% of the total. These for-profit care institutions are mainly run by enterprises, preschool education groups, kindergartens, and individual businesses. The rest are non-profit nurseries that are mainly run by social groups and community service centers, accounting for 31.79% of the total. Inclusive nurseries pilot that are run by enterprise employers in the workplace account for 1.91% of the total (details are shown in Table 8). Of the surveyed institutions, 22.40% said that their main business content was single early education services, including courses for parents and children and interest classes; 6% of the institutions provided a single early childcare service, and 27.60% of the institutions provided simultaneous early education and early childcare services. In addition, some institutions mainly provide other services, accounting for 44%, such as providing guidance or training courses for parents, or training related to the maternity period, as well as real-time home education services (details are shown in Table 9).

**Table 8:** The organization type and sponsor of served institutions.

**Organization type**	**Public**	**Non public**		
Sponsor of the organization	Local government	Enterprises preschool education groups kindergartens individual businesses	Social groups community service centers	Business employer
Proportion	17.86%	48.44%	31.79%	1.91%

**Table 9:** The service content of surveyed institutions.

**Service content**	**Proportion**
Single education service	22.40%
Single care service	6.00%
Both education and care service	27.60%
Other services	44.00%

Finally, with regard to the characteristics of supply, the first is the type of ECCE services provided by childcare institutions. Survey data show that the proportion of institutions providing full-time day care services is relatively high, accounting for 30.70%, followed by half-day care services, accounting for 24.80%, and other services such as temporary day care, accounting for 10.90%. The second is about the distribution of nursery employees in the province and the staffing of ECCE institutions. On the one hand, among all full-time employees, caregivers account for 64.21%, and most full-time employees work in kindergarten classes. The distribution of full-time ECCE employees in various types of childcare institutions is shown in Table 10. On the other hand, the survey data also show that at present, only 62% of institutions are fully staffed, equipped with heads of institutions, professional teachers, healthcare personnel, and other service personnel, while 32.60% of institutions lack healthcare personnel. Only 30.50% of institutions require relevant academic certificates for their managers, professional teachers, and healthcare personnel. The third is about the safety and health conditions of childcare institutions. In terms of institutional environment and facilities, the safety and health conditions of market-oriented institutions are generally uneven and often inferior to those of public institutions. Fourth, research shows that ECCE service charges vary between different types of institutions (Table 11), different organizers (Table 12), and different regions (Table 13), and the charge gap is relatively large. For instance, public ECCE institutions and childcare centers attached to kindergartens charge less; the average monthly charge is less than 1,000 yuan/month/student. However, the monthly average of other types of institutions is higher than 1,000 yuan/month/student.

**Table 10:** Number of employees in nursery institutions in Sichuan Province in 2020.

**Type of mechanism**	**Nursery institution**	**Kindergarten classes**	**Family care center**	**Early childhood education institutions**	**Total**
Number of employees	12,901	696,686	362	1,961	84,910
Number of caregivers among employees	8,258	44,804	280	1,187	54,529
Proportion of caregivers among employees (%)	64	60	77	61	64

**Table 11:** Charges of different types of ECCE institutions in Sichuan Province.

**Organization category**	**Nursery institution**	**Kindergarten classes**	**Family care center**	**Early childhood education institutions take classes**
Average monthly charge (¥)	1,885.81	860.44	1,459.31	1,787.48

**Table 12:** Charges of ECCE institutions of different organizers in Sichuan Province.

**Organizers**	**Public sector**	**Private sector**	**Cooperation between the public and private sectors**	**Enterprises and institutions office**	**Others**
Average monthly charge (¥)	487.70	1,150.21	941.87	1,092.79	1,312.59

**Table 13:** Charges of ECCE institutions in different cities of Sichuan Province.

**City**	**Average monthly**	**City**	**Average monthly**
	**charge (¥)**	**charge (¥)**
Chengdu	1,994.00	Yibin	824.58
Bazhong	1,207.08	Leshan	800.03
Luzhou	1,075.94	Liangshan	781.28
Ganzi	1,055.56	Zigong	773.19
Mianyang	1,007.22	Suining	748.92
Deyang	997.58	Neijiang	743.06
ABa	984.03	Guangyuan	738.35
Meishan	962.25	Nanchong	647.59
Panzhihua	854.92	Ziyang	628.37
Ya'an	839.50	Guangan	535.01
Dazhou	528.68		

## Discussion

### Main Findings

Given that many existing studies have discussed the development of ECCE from the perspective of supply or demand separately, the literature lacks a discussion on the matching of supply and demand. Based on the survey data of the Health Committee, this study analyzes the status quo of ECCE services within an analytical framework of ECCE in Sichuan Province, China.

The findings show that there is a mismatch between the supply and demand of ECCE services in Sichuan Province. First, the total supply cannot meet the total demand. Affected by the comprehensive two-child policy, the number of children aged three and younger has increased. With the miniaturization of the family and women's active participation in the labor market, the family's care function has weakened. Therefore, the total demand for ECCE services is high (1 million), while the supply is far from sufficient (270,000). The average enrollment rate is 54.80%. There is clearly also a mismatch between the supply and demand structure. Parents hope that the main body providing ECCE is the public subject (61.40%), and the service they want to receive is care as well as education (65.73%). At present, ECCE is mainly provided by the market (82.14%), and most of these institutions only provide care services (27.60%). In addition, parents have a greater demand for family ECCE guidance services (47.70%), and the current market supply is even less. Third, the demand and supply of ECCE professionals, institutional security facilities, the environment, and other aspects are also incompatible. There are also significant differences in the quality of health and safety, staff, service content, and environmental facilities among different institutions. There are many reasons for this mismatch, but the primary reasons are as follows.

First, from the perspective of the industry itself, the ECCE service industry is a newly emerging industry in China. The cognition and concept of social support education have lagged behind for some time, and the public's understanding of this industry is still very limited, resulting in the sluggish development of the industry as a whole. Second, from the government level, the top-level design of ECCE services is not perfect, the access standard system and the related policy system and supporting laws and regulations have not been established, and there are problems in ECCE service delivery, such as the lack of the overall management of various departments and unclear management subjects. This means that the industry itself lacks corresponding supervision and has a trend of savage growth. Third, from the perspective of demand, the demand for ECCE is relatively strong. While people hope to take care of themselves at home as much as possible, they also hope that the government can provide relevant services. Therefore, they are flocking to the early education services of public institutions, and are not very optimistic about market-oriented institutional services, which means that there is a structural imbalance in the demand for service institutions. Fourth, from the perspective of supply, the resource base of urban ECCE service supply in Sichuan Province is insufficient. Although the market-oriented ECCE service supply has increased in the last 5 years, demand still exceeds the supply of ECCE services. Driven by interests, ECCE services have been gradually marketized. However, the institutions themselves may not accurately grasp the content and form of ECCE services, resulting in a mismatch between supply and actual demand. In addition, market-oriented institutions aim to make profits. When the profit-seeking, admission, and regulatory standards of ECCE services are missing, the service subjects tend to reduce the service standards to control costs. Therefore, it is more difficult to guarantee the physical and mental safety and health of infants. Therefore, parents' demand for quality ECCE services cannot be met.

### Policy Implication

In order to further promote the development of ECCE in China to better meet the childrearing needs of women and families, efforts should be made in several respects.

First, at the state level, measures should be taken to promote the nature of ECCE supply change toward being more inclusive (49, 50). The government should regard ECCE services as public services in terms of people's livelihoods, and provide more funding for public care and education services for children aged three and younger. It is also necessary to actively explore how to participate in the supply of inclusive ECCE in conjunction with various sectors, especially education, health, community, women's federations, and childcare institutions.

Second, at the community level, communities should be encouraged to provide more ECCE services, so as to enhance the accessibility of ECCE services (51). It is suggested that “community and proximity” care services be explored. Real estate developers should rationally plan ECCE and construct ECCE facilities in newly-built housing estates, while the original communities could make use of the existing resources to set up a public community infant care network equipped with more comprehensive and responsible teaching staff, and provide full-time, part-time, or time-based flexible and diverse services.

Third, at the institutional level, ECCE should be promoted toward the direction of integration of care and education (52). Institutions should speed up the development of scientific standards for infant and childcare. Training bases should be established between universities and institutions to provide more practical opportunities for students in relevant specialties.

Finally, measures should be taken to promote the development of high-quality ECCE supply. Institutions should formulate clear institutional arrangements to ensure the implementation of rules and regulations, such as service objects, service standards, employment requirements, site facilities, and supervision mechanisms. The ECCE service industry should be urged to strengthen self-discipline awareness and gradually purify the nursing service market environment. Institutions also need to provide differentiated services according to the actual demand direction, so as to be more effective in the realization of the allocation of resources and to more accurately satisfy demand.

### Strengths and Limitations

In this study, we analyze the situation of early childhood care and education services in Sichuan province in China through an analytical framework of matching the demand and supply. Our contribution is reflected in two ways. First, this study establishes an analytical framework to assess China's ECCE by matching supply and demand. Second, this study takes official survey data as a solid foundation to analyze the ECCE situation of Sichuan Province, and thereby provides data support for the conclusion of the analysis, ensuring that the research results are more reliable and accurate. However, there are two main limitations of this paper. One is that due to data limitations, this study only uses Sichuan data to analyze the current situation in Sichuan, and lacks analysis of the supply and demand situation in the country. The other is to analyze the current status of ECCE services only from the perspective of supply and demand, without in-depth analysis of other influencing factors.

## Conclusion

In general, this study analyzes the situation of early childhood care and education services in Sichuan through an analytical framework of matching the demand and supply. The findings reveal a mismatch between demand and supply of early childhood care and education services. This gap is likely to have a negative impact on children's self-development, family stability, and even the construction of national early childhood care and education service system. So that measures must be took for improving the mismatch between demand and supply are provided at the state, community and institution levels. It is beneficial for children's self-development, family stability, and even the construction of national early childhood care and education service system in China. In the future, it is best for relevant research to use national data as much as possible to increase universality, and further explore the influencing factors of supply-demand imbalance.

## Data Availability Statement

The original contributions presented in the study are included in the article/supplementary material, further inquiries can be directed to the corresponding author.

## Author Contributions

FP and BY: conceptualization, writing–original draft, and software. XZ: data curation, supervision, and writing–review and editing. YT: methodology and writing–review and editing. All authors contributed to the article and approved the submitted version.

## Funding

Full-Time Postdoctoral Research and Development Fund Project of Sichuan University, Research on the Accurate Configuration of Medical Public Services Empowered by Smart Technology in the Post-epidemic Era (skbsh2020-05) and Independent project of School of Public Administration of Sichuan University, Research on the Accurate Supply of Medical Public Services Empowered by Big Data in the Post-epidemic Era (2020Ziyan-gongguan05). The 2020 Project of the Social Development and Social Risk Control Research Center of Sichuan Philosophy and Social Sciences Key Research Base, Research on Accurate Community Emergency Management Under the Background of Big Data (SR20A09) and the 2021 Dangshi-Dangjian Project of the Central Universities Basic Research Funds for School of Public Administration of Sichuan University, the Development History and Future Prospects of Ethnic Higher Education under the Leadership of the Communist Party of China (2021DSDJ007).

## Conflict of Interest

The authors declare that the research was conducted in the absence of any commercial or financial relationships that could be construed as a potential conflict of interest.

## Publisher's Note

All claims expressed in this article are solely those of the authors and do not necessarily represent those of their affiliated organizations, or those of the publisher, the editors and the reviewers. Any product that may be evaluated in this article, or claim that may be made by its manufacturer, is not guaranteed or endorsed by the publisher.
